# Targeting self-renewal pathways in cancer stem cells: clinical implications for cancer therapy

**DOI:** 10.1038/oncsis.2015.35

**Published:** 2015-11-30

**Authors:** A Borah, S Raveendran, A Rochani, T Maekawa, D S Kumar

**Affiliations:** 1 Bio Nano Electronics Research Center, Graduate School of Interdisciplinary New Science, Toyo University, Kawagoe, Saitama, Japan

## Abstract

Extensive cancer research in the past few decades has identified the existence of a rare subpopulation of stem cells in the grove of cancer cells. These cells are known as the cancer stem cells marked by the presence of surface biomarkers, multi-drug resistance pumps and deregulated self-renewal pathways (SRPs). They have a crucial role in provoking cancer cells leading to tumorigenesis and its progressive metastasis. Cancer stem cells (CSCs) are much alike to normal stem cells in their self-renewal mechanisms. However, deregulations in the SRPs are seen in CSCs, making them resistant to conventional chemotherapeutic agents resulting in the tumor recurrence. Current treatment strategies in cancer fail to detect and differentiate the CSCs from their non-tumorigenic progenies owing to absence of specific biomarkers. Now, it has become imperative to understand complex functional biology of CSCs, especially the signaling pathways to design improved treatment strategies to target them. It is hopeful that the SRPs in CSCs offer a promising target to alter their survival strategies and impede their tumorigenic potential. However, there are many perils associated with the direct targeting method by conventional therapeutic agents such as off targets, poor bioavailability and poor cellular distribution. Recent evidences have shown an increased use of small molecule antagonists directly to target these SRPs may lead to severe side-effects. An alternative to solve these issues could be an appropriate nanoformulation. Nanoformulations of these molecules could provide an added advantage for the selective targeting of the pathways especially Hedgehog, Wnt, Notch and B-cell-specific moloney murine leukemia virus integration site 1 in the CSCs while sparing the normal stem cells. Hence, to achieve this goal a complete understanding of the molecular pathways corroborate with the use of holistic nanosystem (nanomaterial inhibition molecule) could possibly be an encouraging direction for future cancer therapy.

## Introduction

Cancer remains one of the deadliest diseases affecting large number of people worldwide every year. Even after profound cancer treatments, cancer relapse and drug resistance are reported. In the past decade, underlying cause discovered to be associated with tumor recurrence, metastasis and chemoresistance are a relatively small population of stem cells inhabiting each adult tissue called as the cancer stem cells (CSCs). These stem cells in the long run have the opportunity to accumulate the mutations required for malignant transformation owing to their unlimited division potential. These cells were first identified by Bonnet and Dick (1997)^1^ in acute myeloid leukemia and following their findings many other groups have identified these cells in various solid tumors of brain,^2^ breast,^3^ pancreas,^4^ prostate^5, 6^ to name a few. CSCs display certain properties such as high expression of drug efflux transporters, abnormal cellular metabolism, deregulated SRPs, acquisition of epithelial-mesenchymal transition and extensive DNA-repair mechanisms.

**Figure 1:**
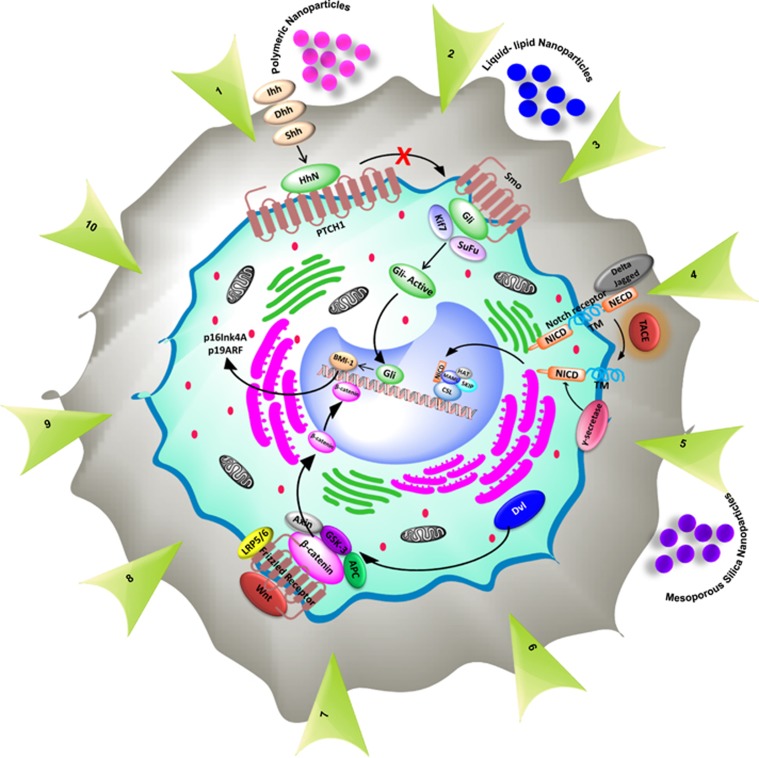
Targeting strategies in self-renewal pathways in CSCs including their pharmacological antagonists and different nanoparticles used for formulation. (1) Hh ligand Inhibitors (2) GLI Antagonists (3) SMO Inhibitors (4) Anti-DLL4 Antibodies (5) γ –Secretase Inhibitors (6) MAML Inhibitors (7) Anti-FZD Antibodies (8) Wnt Ligand inhibitors (9) Wnt Transcription Complex Inhibitors (10) HDAC Inhibitors.

Self-renewal is one of the important properties employed by the CSCs to maintain the proliferating capacities. As genetic and epigenetic changes might have a role in the unrestrained growth, invasion and acquired resistance in cancer cells, it is implicated that epigenesis may accord deregulation of self-renewal pathways (SRPs) in CSCs. There are number of signaling pathways functioning in the normal stem cells, which have assigned roles in the early embryogenesis-like cell proliferation, cell differentiation, cell fate, cell polarity and so on and are under strict regulation. In CSCs, these SRPs when deregulated lead to extensive cell proliferation and may be considered an early event in the process of carcinogenesis. Extensive experimental evidences have revealed Hedgehog (Hh), Wnt, Notch and B-cell-specific moloney murine leukemia virus integration site 1 (BMI1) pathways to be the key players in maintaining the proliferating capacity of CSCs and activated in most of the solid tumors.^7^ Among other signaling proteins such as phosphatase and tensin homolog,^8^ bone morphogenetic protein and transforming growth factor beta are also of specific interest as they too control self-renewal and cell differentiation in various tissues and are additionally implicated in tumorigenesis. Recent investigations of targeting the signaling pathways in CSCs have found to be of prime interest. This review focuses on several aspects of major SRPs, which are found to be upregulated in CSCs and certain novel strategies to target these pathways by nanodrug-delivery platforms for the prevention of tumor relapse and chemoresistance (Figure 1).

## Self-renewal pathways in CSCs

CSCs make up a minor fraction of the tumor tissues. It acquires a heterogeneous phenotype and can maintain tumor formation at a high degree. Apparently, it is seen that the CSCs share common attributes with the normal stem cells, for instance, self-renewal and differentiation capacity. However, there exist fine-drawn differences between CSCs and normal stem cells for using the same pathways. The molecular mechanisms underlying these phenomena of CSCs hijacking the SRPs of normal stem cells for its own maintenance though remains vague. In the following sections, we are going to review the potential pathways, which are implicated in the CSCs self-renewal activity and tumor initiation through immense experimental findings.

## Hh pathway

It is known that the Hh pathway helps in controlling cell growth, tissue patterning, morphogenesis^9^ in animal development. The Hh family of proteins has at least three Drosophila Hh gene homologs in vertebrates: Sonic Hh (SHh), Desert Hh and Indian Hh, among which SHh is the most widely used one. The Hh is a 400–460 amino-acid long precursor protein. The (HhN) amino-terminal domain works as a signaling molecule, whereas the carboxy-terminal domain (HhC) has an auto-catalyzing Hint module. The signaling cell releases the Hh protein through a committed transmembrane receptor called the Dispatched. This happens only after the amino terminal of the Hh protein is being palmitoylated by Rasp/Skinny located in endoplasmic reticulum.^10^ The modified Hh protein binds to its 12 transmembrane receptor known as the Patched (Ptc) and initiates the signaling process. In Hh pathway, a seven-pass transmembrane receptor named Smoothened (Smo) activation is necessary for further signaling process. In the absence of Hh, the Ptc prevents Smo from being located to the primary cilium and its catalytic activity. However, when Ptc is bound by Hh ligand, the inhibitory effect of Ptc on Smo is rendered inactive. Smo now activates the Gli family of transcription factors to carry out the downstream signaling process. Without Smo activation, Gli is maintained in a complex with Suppressor of Fused, which is a negative regulator of Hh signaling. Upon Smo activation, Gli is dissociated from Suppressor of Fused-Gli complex for nuclear translocation to promote the transcription of Hh targeted genes namely patched, cyclin (D/E). In mammals, there are three types of Gli transcription factors Gli1, Gli2 and Gli3 of which Gli1 and Gli2 are activators and Gli3 acts as a repressor. The loss of Suppressor of Fused results in the activation of Hh signaling, which indicates its central role in the repression of the pathway.^11^

The Hh signal transduction pathway components tightly control embryonic development, and also expressed in postnatal and adult tissues, where these components have assigned roles in the maintenance of stem cells, tissue repair and regeneration. Hence, defects in Hh signaling may affect at the embryonic and later stages of life in humans.^12^ Many human congenital diseases have been associated with Hh signaling defects such as holoprosencephaly in which there is loss of one copy of SHh.^13^ Mutations in Ptc1 result in a rare autosomal genetic form of basal cell carcinoma also known as the Gorlin syndrome.^14, 15^ Increasing evidence have widely supported the fact that dysregulated Hh signaling is present in majority of the human cancers today, which includes brain tumors, melanomas, leukemia's, gastro-intestinal, malignancies of the breast, ovary, prostate and pancreas.^16^ However, in most of these cancers mutation of Hh pathway components is not the only basis for its aberrant activation, but rather has been caused by high expression of Hh ligands.^17, 18^ Experimental evidences in the past have confirmed the presence of CSCs in most of the human tumors and the self-renewal property of these cells has been attributed to Hh signaling.^19, 20, 21, 22, 23^ Hh signaling maintains the self-renewal capacity of the malignant clone, which was demonstrated in mouse models of chronic myeloid leukemia.^20, 23^ Hh signaling is also under epigenetic regulation in CSCs mainly the Gli transcription factors. As Gli1 and Gli2 are acetylated, their deacetylation mediated by Histone deacetylase (HDAC) complex promotes Hh pathway activation. Downregulation of Gli1 is mediated by miR-324-5p, and subsequent loss of miR-324-5p have led to neoplastic transformation into medulloblastoma.^24^ Ptc and Gli1 proteins were seen to be highly expressed in ovarian cancer patients as reported by Liao *et al.*^25^ The authors in this study also observed that there was a significant overexpression of SHh mRNA in the patient's tumor tissues. It is also affirmed that Hh signaling has an active role in the progression of prostate cancer; however, there is paucity of the precise mechanism involved in its abnormal signaling. Sheng *et al.*^26^ have reported a loss-of-function mutations in Suppressor of Fused, in most of the prostatic tumor tissues. Other independent studies carried out by groups have presented with data that there is a ligand-dependent paracrine or autocrine Hh signaling in prostate tumors.^27, 28^ Hh signaling is also found to regulate self-renewal in normal and mammary CSCs acting in concert with BMI pathway as investigated by Liu *et al.*^29^ in their *in vitro* and *in vivo* studies.

## Notch pathway

Notch signaling is a developmental pathway in multicellular organisms involved in cell fate decisions and pattern formation during embryogenesis.^30^ Post-translational modifications result in the formation of a heterodimeric NECD (notch receptor comprising of an extracellular domain) and TM-NICD (transmembrane-intracellular domain) inserted in the plasma membrane of a signal-receiving cell. Once a ligand for example, Delta (DLL1, DLL3, DLL4) and Jagged (jag1, jag2) binds to the notch receptor, the TNF-alpha ADAM metalloprotease-converting enzyme mediates the cleavage of NECD from TM-NICD. The NECD-ligand complex is endocytosed/recycled in the signal-sending cell by Mind Bomb ubiquitination, whereas in the signal-receiving cell the γ-secretase enzyme cleaves TM-NICD complex, releasing NICD. It further proceeds into the nucleus and associate with the CSL (centromere-binding factor 1/Suppressor of hairless/Lag1) transcription complex. This CSL-NICD complex now subsequently activates the notch target genes: Hairy and enhancer of split family, p21 and Myc.

Apart from regulating cellular communication in embryogenesis, it also helps in stem cell growth and differentiation. Studies have elucidated the pathological role of notch pathway in human malignancies going from T-cell acute lymphoblastic leukemia (T-ALL)^31^ to breast cancer^32, 33^ and others where inappropriate activation of the pathway that led to uncontrolled proliferation, restricted differentiation and prevents apoptosis in the cancer cells. Of late, a mere reason of focusing on notch pathway in recent years is due to the identification of a distinct cellular hierarchy in human acute myeloid leukemia^1^ and other solid tumors.^2, 3^ This cellular hierarchy is the CSCs, which maintains the tumor and recapitulates the features of normal stem cells. Notch pathway is one of the developmental pathways active in this subset of CSCs, which maintains the self-replication and differentiation decisions. A significant evidence of the Notch pathway, that it is related for the survival of CSCs, came from the independent studies conducted by Farnie and Clarke;^34^ Sansone *et al.*^35^ Farnie and Clarke reported the role of aberrant notch signaling as one of the factors involved in early breast cancer. Studies by Gustafasson *et al.*^36^ have indicated that the notch and hypoxia response factor HIFα interacts with each other to assist the outset of a stem cell phenotype and its survival in hypoxic environment. Based on these findings, Sansone *et al.*^35^ carried out various studies to report that the expression of notch-3 is being controlled by the 66k-Da isoform of the Src homology 2 domain-containing gene (p66Shc), which gets induced in a breast cancer cell line when exposed to a hypoxic environment also leading to the survival of mammary gland progenitor cells. Notch signaling also has an oncogenic role in T-ALL where Notch 1 was identified to be involved in t (7; 9)(q34;q34.3) chromosomal translocation to bring out the disease outcome.^37^ Subsequent studies have brought newer insights to the role of Notch in human T-ALLs, with discovery of two types activating mutations within Notch 1.^38^ One mutation was in the extracellular hetero-dimerization domain, a change in the amino-acid sequence leading to ligand-independent metalloproteinase cleavage site S2, whereas the second involved Notch 1 proline, glutamic acid, serine, threonine sequence domain. These mutations were reported to be present in 50% of human T-ALLs.^38^ Notch 1 is also shown to have an elevated expression in pancreatic CSCs compared with non-pancreatic CSCs.^39^ In pancreatic cancer, notch pathway maintains the epithelial cells in a progenitor state, acquiring epithelial-mesenchymal transition phenotype leading to tumor growth, invasiveness and metastasis.^40, 41^ Emerging evidences show that the resistance of pancreatic cancers toward several chemotherapeutic measures is due to activated Notch signaling, although underlying mechanism still remains elusive.^41, 42^ These studies provides the rationale to develop targeted therapies, which will interfere with notch signaling in human malignancies.

## WNT pathway

The Wnt signaling pathway is an ancient and evolutionary conserved developmental pathway, which controls stem cells and determines cellular fate during development. The Wnt family is a group of 19 glycoproteins in humans involving a complex mechanism of signaling phenomena, with salient functional and biological outcome.^43^ It may lead to much serious pleiotropic pathology when these tightly controlled mechanisms go awry. The Wnt ligand binds to a transmembrane receptor Frizzled and displaces the GSK-3β (glycogen synthase kinase 3 beta) from the adenomatous polyposis coli (APC)/Axin/GSK-3β regulatory complex. However, the absence of Wnt ligand marks the degradation of β-catenin a cell adhesion protein and transcription regulator in APC/Axin/GSK-3β and casein kinase1 destruction complex^44, 45^ through the beta transducing repeat containing E3 ubiquitin protein ligase pathway. Once Wnt ligand binds to its receptor the pathway is turned on and brings the co receptor low-density lipoprotein receptor related protein 5/6 to the vicinity of the Wnt bound Frizzled complex. This activates downstream component Disheveled by sequential phosphorylation, polyubiquitination, polymerization and finally stabilizing β-catenin.^46^ β-catenin now translocate to the nucleus where it associates with T-cell factor/lymphoid-enhanced factor family of transcription factors, and recruits other co-activators such as cAMP response element-binding protein, p300,^47, 48^ Bcl9^49^ and Pygopus.^50^ This ultimately leads to transcription of target Wnt genes: survivin, cyclin D and c-myc.

The relevance of Wnt signaling in human cancers was perhaps best well known for its role in colon cancer where the healthy colonic epithelia accumulates mutation in specific genes such as APC, β-catenin, K-ras and p53.^51^ Morin PJ *et al.*^52^ had carried out genetic studies in four different kinds of APC mutants and analyzed that the presence of APC mutations in colorectal cancer also leads to defective downregulation of β-catenin and Tcf-4 transcriptional activity. There are numerous mechanisms that can drive the aberrant Wnt/β-catenin signaling, leading to cancer formation in a mutually exclusive manner. In certain colorectal cancers, there is a probability of finding an exclusive catenin (cadherin-associated protein) beta 1 mutation when APC mutations are lacking.^53, 54^ This was also supported by the conclusive evidence, which came from the studies of Mirabelli-Primadehl *et al.*^55^ regarding the role of β-catenin mutations in colorectal cancers. Hepatocellular carcinoma^56^ and endometrial ovarian tumors^57, 58^ were also found to possess catenin (cadherin-associated protein) beta 1 mutations, which led to aberrant nuclear accumulation of β-catenin. A vast majority of the colorectal tumors harbor APC mutations, which may lead to the constitutive activation of β-catenin^59, 60, 61^ Like Hh and Notch, Wnt/β-catenin signaling too has an important role in embryogenesis and regulates cell proliferation and lineage differentiation in many tissues.^62^ In adults, Wnt signals are basically involved in stem cell renewal especially in intestinal crypts,^63^ hair follicles^64^ and bone growth plate.^65^ As Wnt signaling has a notable role to play in stem cell proliferation and differentiation, its disruptions will certainly affect stem cell function with serious implications for malignancy. Consistent findings have shed light to the fact that β-catenin is present in a variety of CSCs settings^66, 67, 68^ including colon,^69^ cutaneous CSC^70^ and also HSC.^71^ Among all these CSCs, colon CSCs were found to have a very high concentration of β-catenin, which contributes to its stemness, in part orchestrated by the microenvironment finally giving rise to drug resistance and also metastasis.^69^ Wnt signaling has been also shown to be responsible for epithelial-mesenchymal transition^72^ in tumors as a result of high concentration of β-catenin in the nucleus.^73^ This leads to the arrest of tumor cell division and acquiring mesenchymal markers like fibronectin^74^ while retaining the self-renewal capacity, a characteristic feature employed by the CSCs.

## BMI1 pathway

The BMI1 pathway is one of the proto-oncogenic signaling pathways like Hh, Notch and Wnt involved in the differentiation and self-renewal mechanisms of stem cells persistently.^75^ The BMI1 belongs to the Polycomb group of gene family, well-known epigenetic gene silencers, targeting the p16 and p19Arf locus^76^ both of which suppresses cell proliferation. Human BMI1 gene comprises of 10 exons and is localized on chromosome 10.^77^ BMI1 gene encodes a 324 amino-acid long protein with a predominant nuclear localization comprising of a *N*-terminal RING finger domain and a central helix turn helix motif.^78^ BMI1 affects morphogenesis during embryonic development and in hematopoiesis as reported by van Der Lugt *et al.*^79^ in 1994 with a pervasive expression in almost all tissues. Extensive studies have also reported the association of BMI1 in the initiation of various cancers where BMI1 can cooperate with c-myc and initiate the disease.^80^ Its expression was found to be highly upregulated in acute myeloid leukemia,^81^ cancers of the lung,^82^ ovaries,^83^ breast^84^ and neuroblastoma.^85^ It is noted that CSCs are highly enriched with BMI1, and seen to be co-expressive with stem cell markers, CD133 and CD44, in most of the tumor CSC population.^86, 87, 88^ Zhang *et al.*^89^ in their study asserted that epithelial ovarian cancers arise from a population of tumor-initiating cells with the CD44- and CD117-positive marker phenotype along with the expression of BMI1 and others such as Notch 1, ATP-binding cassette sub-family G member 2, Nanog, Nestin and Oct-4. The expression of these markers led to chemoresistance and exacerbated the disease condition. Cui H *et al.*^85^ reported BMI1 to be overexpressed in human neuroblastoma primary tumors and cell lines, cooperating with MYCN gene in transforming the benign S-type neuroblastoma cells. Prostate cancer cells too have a heightened expression of BMI1 in tumors with Gleason scores of 8 or higher.^90^ Glinsky and colleagues^91^ carried out a microarray analysis in 11 different types of cancer specimens and indicated that the conserved BMI1 driven pathway is engaged in a metastatic behavior of human malignancies along with a stem cell-like expression profile ultimately leading to disease recurrence after therapy. These studies indicate that the overexpression of BMI1 is critical for the maintenance of CSCs in most of the human tumors.

## Targeting strategies to inhibit self-renewal pathways in CSCs

Conventional cancer treatment of chemotherapy and radiotherapy can target only the bulk of sensitive tumor cells, which are in rapidly dividing phase. This therapeutic intervention induces many tumor cells to undergo apoptosis and die, whereas the CSCs survive this process by remaining in G^0^ phase and give rise to 'second-line tumors' with acquired resistance.^92, 93, 94^ Henceforth, current cancer research is focused toward targeting these CSCs and it has become essential to develop novel therapeutic approaches to prevent cancer recurrence and emergence of drug resistance. Even though tremendous research has been carried out to eliminate the CSCs, but efficient modalities to target the SRPs in CSCs have been gaining prime focus in recent years. During and after the treatment period CSCs maintain their self-renewal and differentiation capacities by activating the embryonic signaling pathways. The Hh, Notch, Wnt and BMI1 maintains the proper functionality in normal stem cells but a deregulated behavior in these pathways, owing to some alterations in the genes encoding the signaling molecules is observed in CSCs and also have been found in human tumor samples clearly stating their role in tumor development and maintenance.^95, 96^ As normal stem cells and CSCs share similarities in the signaling pathways, it would be extremely important while designing drugs to understand the complex biology of these pathways to destroy the CSCs and selectively sparing the normal stem cells.

## Drugs targeting self-renewal pathways

Cyclopamine, a plant derived teratogen binds and deactivate Smo which is otherwise being suppressed by Ptc. Targeting the Hh pathway using cyclopamine was shown by Taipale *et al.*^97^ where they suggested that Hh pathway related tumors associated with Ptc mutations might respond well to treatment with cyclopamine. As cyclopamine is a steroidal compound, it affects the activity of Ptc by blocking its sterol-sensing domain.^98, 99^ Bar EE *et al.*^100^ conducted a study on cyclopamine-mediated inhibition of Hh pathway in glioblastoma CSCs, and observed a significant 40–60% decrease in growth of adherent glioma cell lines with high Gli1 expression and no new neurospheres formed. Apart from cyclopamine, another synthetic small molecule inhibitors of Smo, GDC-0449 identified by Genentech was shown to inhibit the Hh pathway activity in metastatic basal cell carcinoma (ClinicalTrials.govnumber, NCT00607724).^101^ Oral administration of GDC-0449 was given to 33 patients with advanced basal cell carcinoma for a median duration of 9.8 months and reported two complete responses and 16 partial responses.^101^ GDC-0449 was also shown to have its inhibitory effect in medulloblastoma, pancreatic cancer but its effect is more prominent in advanced basal cell carcinoma. Several other small molecule Smo antagonists, which are investigated clinically include IPI-926,^102^ BMS-833923 (Clinical trials.govnumber, NCT00884546), PF-04449913 (Clinical trials.govnumber, NCT00953758), LDE-225.^103, 104^ However, there may be resistance to these molecules over a period of time due to point mutations in Smo. Hence, targeting the SHh ligand and the downstream components such as Gli transcription factors by small molecules namely Robotnikinin^105^ and HPIs 1-4,^106^ GANT58,^107^ GANT61,^107^ respectively, is a promising approach to prevent tumor relapse and metastasis. In addition to chemical compounds used for the treatment of human cancer, researchers have also considered the use of dietary chemopreventive agents known as nutraceuticals for targeting the Hh signaling such as Resveratrol,^108^ Curcumin^109^ and epigallocatechin-3-gallate,^110^ which have been experimentally shown to inhibit Hh signaling in prostate cancer, medulloblastoma and chondrosarcoma, respectively.

Most of the agents that have been developed to inhibit notch signaling are designed to target notch ligands, notch receptors, ligand receptor binding, γ-secretase-mediated cleavage and transcriptional nuclear complex. γ-secretase inhibitors are small molecule agents, which are widely studied, as notch activation largely depends on γ-secretase activity and is a promising target. A number of clinical trials on γ-secretase inhibitors is well indicated to inhibit notch signaling in many cancers, for example, T-ALL, central nervous system malignancies,^111^ breast cancer.^112^ MK0752, one of the potent γ-secretase inhibitors in clinical development was shown to inhibit notch signaling in majority of human T-ALL.^113^ Another γ-secretase inhibitor PF-03084014 was shown to inhibit Notch activity in T-ALL cell lines by Wei P *et al.*^114^ Apart from targeting the γ-secretase activity, notch ligand-inhibiting agents specially DLL4 monoclonal antibodies, for example, OMP-21M18 are in clinical development, designed for patients diagnosed with colon cancer, pancreatic cancer and small cell lung cancer.^115^ DLL4, specific notch ligand for embryonic vascular development and arteriogenesis^116, 117^ when blocked by a selective antibody-impeded tumor growth in several solid tumor models.^118^ Other agents that inhibit notch signaling in cancer include mastermind-like peptide inhibitors, which interferes with the notch nuclear co-activator mastermind-like protein, a part of the Notch transcriptional complex^119^ and notch soluble receptor decoys.^120^ Also, the use of natural compounds such as genistein,^121^ sulforaphane,^122^ quercetin^123^ owing to their relative low toxicity was seen to inhibit notch activity in tumor cells or in CSCs.

Agents that can inhibit Wnt signaling, currently under investigations, employ strategies to target receptor/ligand interactions, cytosolic and nuclear signaling components. One of the approaches to inhibit receptor ligand interactions is to target the Frizzled family of receptors by using antibodies. Studies have been carried out using a humanized antibody against Frizzled 10 for patients with synovial sarcoma.^124^ *In vitro* studies revealed that synovial sarcoma cells were suppressed by the polyclonal antibody in mediating antibody dependent cell-mediated cytotoxicity against the Frizzled 10 receptor overexpressed cells.^124^ Monoclonal antibodies targeting the Wnt (1–2) ligands have also disclosed the inhibition of Wnt signaling in colon cancer^125^ and human melanoma.^126^ Disheveled protein is one of the key cytosolic signaling components in the Wnt pathway that associates extracellular signals to its downstream components. Disheveled could be a therapeutic intervention in inhibiting the Wnt pathway for cancer therapy. Compounds that have been preclinically tested in this direction include FJ9^127^ and NSC668036.^128^ One of the critical steps in the activation of Wnt signaling is the interaction of β-catenin with the T-cell factor/lymphoid-enhanced factor transcription factors, and recruits a myriad of co-activators such as cAMP response element-binding protein, p300 to name a few.^47^ These co-activators represent potential targets to interfere with the β-catenin/transcription factor stabilization complex. ICG-001 a small molecule inhibitor^129, 130^ (Institute for chemical genomics) was developed in this direction to target these co-activators.

BMI1 has no enzymatic function hence traditional drug discovery approaches to target this protein remains a challenge. However, the use of HDAC inhibitors to suppress the expression of BMI1 and its downstream components was recently shown by Bommi *et al.*^131^ in human breast cancer. The HDAC inhibitors such as sodium butyrate and valproic acid were investigated in the study where the compounds seem to inhibit BMI1 activity through a transcriptional mechanism repressing the polycomb complexes. Another drug artemisinin and its derivatives having antimalarial activity were shown to have inhibition on cancer cell growth and angiogenesis. This drug was investigated to check its inhibitory role in regulating BMI1 expression both in protein and transcript levels in nasopharyngeal carcinoma cells.^132^ To date, no small molecules have been reported to inhibit BMI1 with competent specificities, although experimental evidences cited above using HDAC inhibitors and artemisinin bring a rationale to develop more agents for therapeutic targeting of BMI 1.

## Prospects of nanodrug targeting

In current cancer treatment strategies, targeted drug delivery is one of the safest ways to target the tumor. To address this issue, nanoparticles have had an important role in delivery of drugs specifically at the designated site at the required concentration, evading immune response without having any off targets within the safety margins. Nanoparticles in the past have received quite unprecedented success as drug-delivery vectors in cancer therapy and diagnosis because of their biophysiological properties and the ability to interact with cells due to the similarity of their size with cellular components.^133, 134, 135^ They can carry multiple payloads owing to their large surface area, multi-functionalized with targeting moieties and controlled drug release.^136, 137^ Taking into account about the multiple advantages of nanoparticles, they can be harnessed to the best of their ability to target the drug-resistant CSCs. Independent studies conducted by researchers have applied nanoparticles to target CSCs in diverse overlapping areas. Lee *et al.*^138^ and Swaminathan *et al.*^139^ in their distinctive studies have made use of nanoparticles as 'beacons' to label CSCs as a diagnostic measure. Nanoparticles were also successfully used to deliver non-druggable anticancer agents to kill the drug-resistant CSCs.^140^ Moreover, nanoparticles in the form of stealthy liposomes were used as therapeutic intervention by Liu *et al.*^141^ to wipe out CSCs and non CSCs selectively. Many groups have recently targeted the CSCs effectively through the use of combination therapy of antibodies and conventional chemotherapeutic drugs against the CSC surface markers CD133^+^^142^ and drug efflux transporters.^143^ Yu *et al.*^144^ in their study eliminated CD133^+^ osteosarcoma CSCs through salinomycin delivery via CD133 aptamer-conjugated PEGylated PLGA nanoparticles. These approaches though have received encouraging results, but still leave plenty of room for improvement. Another approach to target the CSCs, which is the main focus of this review, and have received a lot of attention over the years is the targeting of the SRPs, which are implicated to maintain the self-renewal capacity of the CSCs and involved in tumorigenesis. Till date, SRPs as discussed in the above sections are being targeted directly by the use of small molecule inhibitors, monoclonal antibodies and natural compounds. Although these agents have shown promising results in inhibiting the deregulated pathways in CSCs^145, 146^ there have been certain drawbacks associated such as toxicity, poor water solubility and poor specificity. Hence, nanoformulation of these compounds along with the combination of conventional chemotherapeutic drugs is a holistic approach to inhibit the SRPs in CSCSs.

**Figure 2:**
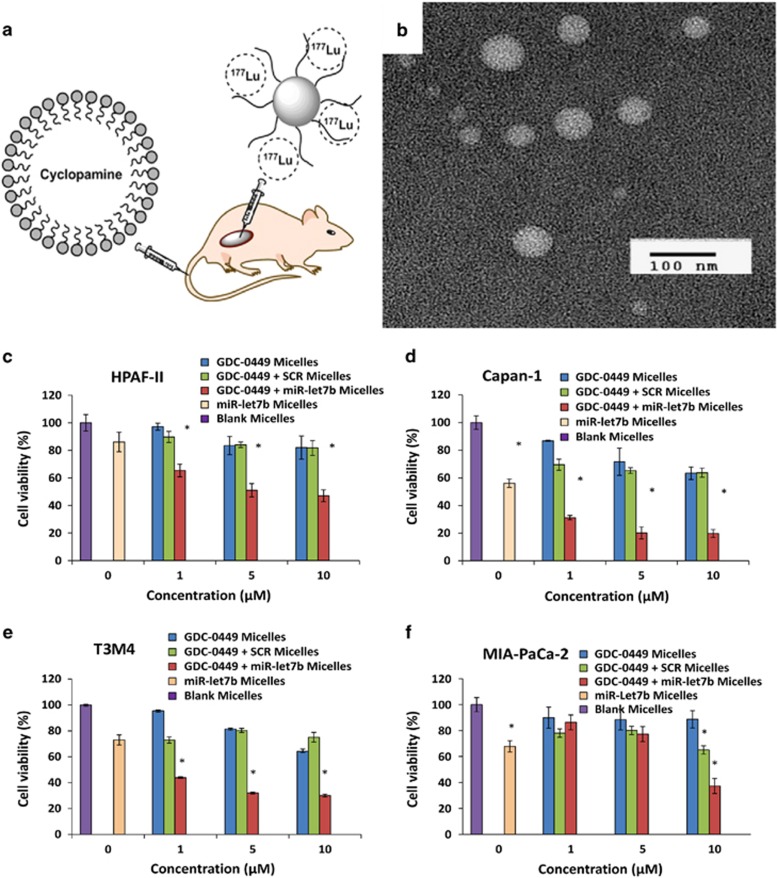
(**a**) Schematic illustration of study design. Radioactive polymeric micelles containing 177Lu were injected intratumorally, and CPA-loaded lipid nanoparticles were injected intravenously. (**b**) Transmission electron microscopy images of CPA- LLP (negative staining). (Reproduced with permission from You J *et al.* 2015). (**c**–**f**) Effect of GDC-0449 and miR-let7b on cell viability in human pancreatic cancer cell line by micelles. HPAF-II, Capan-1, T3M4 and MIAPaCa-2 cells (5000/well) were treated with micelles containing (blue bars) GDC-0449 (0, 1, 5 and 10 μM), (green bars) GDC-0449 and scrambled miRNA, (red bars) GDC-0449 and miR-let7b (10 pmol), (peach bars) miR-let7b alone, and (purple bars) blank for 48 h. Cell viability was measured by MTT assay at the end of incubation period. (Reproduced with permission from Kumar V *et al.* 2015).

Chenna *et al.*^147^ recently have engineered a polymeric nanoparticle encapsulating a small molecule inhibitor, HPI-1 (Hh pathway inhibitor), which was shown to bypass the secondary mutational resistance toward Smoothened antagonists. Hh signaling is seen to be aberrantly active in most of the human cancers, and Smo secondary mutation abrogates the binding of most of the Hh inhibitors. The group addressed this issue by nanoformulating HPI-1 (NanoHHI) that is a potent antagonist of Gli1 and reported that NanoHHI markedly inhibits the growth of mouse medulloblastoma allografts, which harbor a Smo^D477G^-binding site mutation, accompanied by significant downregulation of Gli1 mRNA. Nanoformulation of HPI-1 improved its aqueous solubility and also systemic bioavailability.^147^ The same group further confirmed their studies by using NanoHHI to check the inhibition of Hh signaling in hepatocellular carcinoma (HCC) in an orthotopic model. NanoHHI markedly reduced systemic metastases in HCC cell lines both in vitro and *in vivo* settings. Moreover, it also decreased the population of CD133^+^-expressing HCC cells, considered to be the tumor-initiating cells.^148^ Lim K *et al.* revealed that polymeric nanoparticle formulation of curcumin suppressed the growth of multiple brain tumor cell lines. The authors observed that NanoCurc when administered to brain tumor cell lines in a dose-dependent manner, it led to programmed cell death in addition to depleting CSCs. In their study, microarray analyses disclosed that when medulloblastoma DAOY cells treated with 20 μM curcumin showed 2.4-fold downregulation of Gli1 expression, which is a key effector in Hh signaling. However, notch activity was not seen to be much affected by curcumin treatment in DAOY cells.^149^ A liquid–lipid nanoparticle delivery system has been harnessed in a recent study by You *et al.*^150^ to deliver the Smo antagonist CPA-LLP (cyclopamine) in 4T1 murine breast cancer and Miapaca-2 human pancreatic carcinoma models (Figures 2a and b). The group used a combination strategy of CPA-LLP and core-cross-linked polymeric micelles bound lutetium-177 in the carcinoma models and reported slow tumor growth. Pancreatic ductal adenocarcinoma is characterized with desmoplasia, aberrant Hh signaling and downregulation of tumor suppressor miR-let7b. Desmoplastic environment provides the niche for CSCs. Mahato *et al.*^151^ carried out synergistic treatment of pancreatic ductal adenocarcinoma through co-delivery of Hh inhibitor GDC-0449 and miRNA (miR-let7b) into micelles using methoxy poly (ethylene glycol)-*block*-poly (2-methyl- 2-carboxyl-propylenecarbonate-graft-dodecanol-graft-tetraethylene-pentamine) (mPEG-b-PCC-g-DC-g-TEPA). It was observed that the combination therapy of GDC-0449 and miR-let7b micelles led to reduced cell viability in the different pancreatic cell lines (HPAF-II, Capan-I, T3M4, MIA-PaCa-I) even at low dose concentration of the formulation (Figures 2c–f).

**Figure 3:**
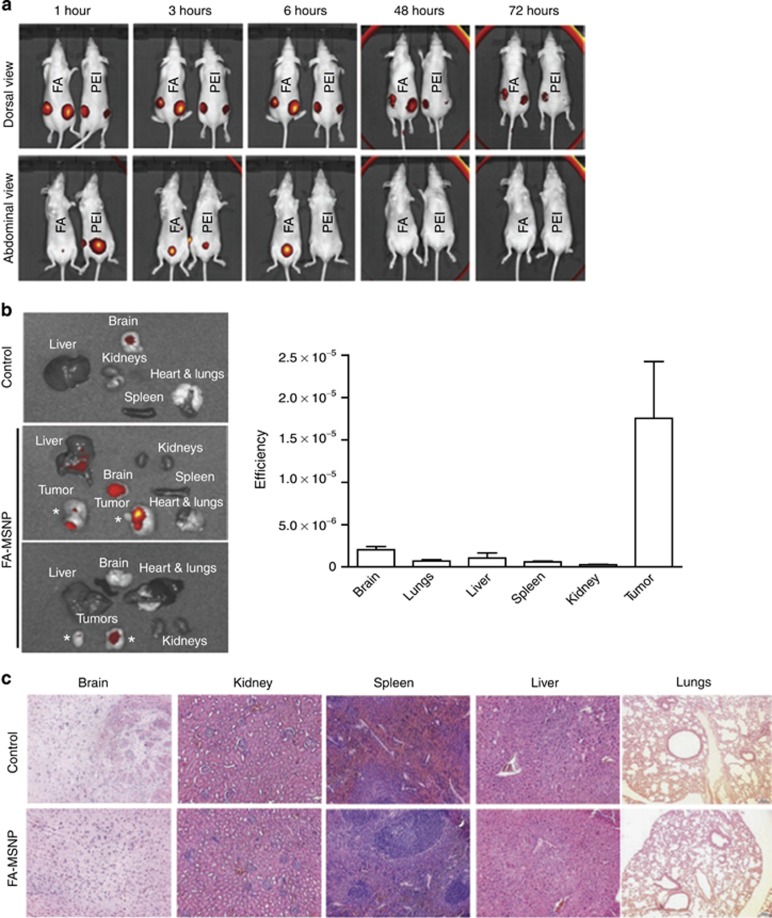
Mesoporous silica nanoparticles (MsnPs) accumulate in the tumors, are biocompatible biodegradable and eliminated through renal excretion. (**a**) *In vivo* imaging of mice injected peritumoral with PEI-MSNPs or folate (FA)-MSNPs. Images of the abdominal area demonstrate accumulation of fluorescence in the bladder, and imaging of the dorsal area show accumulation of fluorescence in the tumors. Time lapse imaging of the abdominal area shows elimination of fluorescence within 48 h after injections (number of animals per group, *n*=4, two tumors per animal). (**b**) *Ex vivo* analyses (left) and quantification of fluorescence intensity (right) in organs from mice injected intravenous (i.v.) with FA-MSNPs. Mice were killed 196 h after injection (*n*=4). Please note the occasional signal from brain tissue, which most likely represents background fluorescence, as it is present also in untreated control animals. (**c**) Histological analysis of the brain, kidney, spleen, liver and lungs of untreated mice and FA-MSNPs-treated mice showed no morphological changes. Mice were killed 192 h after i.v.injection. (Reproduced with permission from Mamaeva V *et al.* 2011.)

Notch signaling is mostly targeted by the use of gamma-secretase inhibitors but its clinical use is hindered by acute after-effects and hence the need for an alternative strategy. A novel approach of delivering the gamma-secretase inhibitors to block Notch signaling was presented by Mamaeva and colleagues using imagable mesoporous silica nanoparticles, which were found to be biocompatible, biodegradable and delivered gamma-secretase inhibitors without any toxic side-effects (Figures 3a–c). The group designed a drug-loaded mesoporous silica nanoparticles of average size centered ~200–350 nm and surface modified with folate (FA) to the outer polyethylenimine layer of the particles. *In vitro* analyses were screened using different breast cancer cell lines (MCF7 (FR-positive), MDA-MB-231, T47D, SK-BR-3, MDA-MB-468). The study revealed the mesoporous silica nanoparticles-mediated delivery of gamma-secretase inhibitors was specific toward the cells and also inhibited Notch signaling. MCF7 cells were reported to have the highest FA-mediated endocytosis due to its surface functionalization. Moreover, *in vivo* studies also supported that targeted gamma-secretase inhibitors delivery-enhanced tumor penetration and retainment at the tumor site as compared with free drug.^152^ Recently Lo *et al.* have designed a small interfering RNA-delivery approach against the enhancer of zeste homolog 2 and Oct-4 genes upregulated in head and neck squamous cell carcinoma using polyurethane-short branch polyethylenimine. The small interfering RNA polyethylenimine constructs used was able to repress epithelial-mesenchymal transition and radioresistance in aldehyde dehydrogenase 1+/CD44+ CSC-like cells, in addition to inhibiting Wnt signaling, which may be involved in the CSCs.^153^

Although these experimental findings are encouraging to target the SRPs through nanoparticle-mediated delivery. However, it is imperative to extend more research in combining the SRPs-targeting therapeutics with nanotechnology-based platforms for a robust cancer treatment strategy for clinical applications.

## Conclusion and future direction

In this review, we have tried to render a picture of the heterogeneous CSCs being implicated to be a cause of cancer relapse, chemo and radioresistance in recent times. Understanding the complex biology behind the survival mechanism of CSCs in solid tumors, deregulation in the SRPs is seen to be one of the prominent reasons for their inevitable existence even after treatment. Despite the availability of small molecule inhibitors used to target the SRPs, a small fraction of them only has been put to clinical application owing to their non-specific toxicity and solubility issues. This could be solved by nanoformulating these compounds, which will overcome their barriers and specifically deliver these molecules to the designated sites. Nanoparticles as mentioned above have been used in recent times to target the CSCs in solid tumors; hence, nanotechnology could also be extended to target the SRPs active in CSCs. As there occurs crosstalks between the different signaling pathways in cancer development and progression, inhibition of one could lead to the downregulation of the others. Nanoparticles could provide a platform to carry multiple pathway inhibitors along with a conventional chemotherapeutic to target the pathways. Although there have been very few reports cited in literature in this direction, comprehending the biology of the pathways combined with the use of wide range of nanoparticles in dispose is a challenging area of research and leaves a futuristic hope for cancer treatment in killing the CSCs.
